# Effects of exogenous protease addition on fermentation and nutritive value of rehydrated corn and sorghum grains silages

**DOI:** 10.1038/s41598-023-34595-w

**Published:** 2023-05-05

**Authors:** João Paulo Santos Roseira, Odilon Gomes Pereira, Tâmara Chagas da Silveira, Vanessa Paula da Silva, Wagner Sousa Alves, Mariele Cristina Nascimento Agarussi, Karina Guimarães Ribeiro

**Affiliations:** Department of Animal Science, Federal University of Vicosa, Viçosa, 36570-900 Brazil

**Keywords:** Applied microbiology, Plant biotechnology

## Abstract

The study objective was to evaluate the effects of the addition of exogenous protease on the fermentation and nutritive value of rehydrated corn and sorghum grain silages during various storage periods. Treatments were applied using a 2 × 6 × 3 factorial combination, with 2 types of rehydrated grains (corn and sorghum), 6 doses of the enzyme (0, 0.3, 0.6, 0.9, 1.2, and 1.5%, based on natural matter) and 3 fermentation periods (0, 60, and 90 days) in a completely randomized design, with 4 replications. The protease aspergilopepsin I, of fungal origin, produced by *Aspergillus niger*, was used. The lactic acid concentration increased linearly as the enzyme dose increased in corn (CG) and sorghum (SG) grain silages, at 60 and 90 days of fermentation. There was an increase in the concentrations of ammonia nitrogen and soluble protein, as well as the in situ starch digestibility in rehydrated CG and SG silages, compared to the treatment without the addition of protease. The addition of 0.3% exogenous protease at the moment of CG ensiling and 0.5% in rehydrated SG increased the proteolytic activity during fermentation, providing an increase in in situ starch digestibility in a shorter storage time.

## Introduction

Rehydrated grain silage is the product resulting from the anaerobic fermentation of mature, ground grains, with reconstituted moisture, in which microorganisms consume water-soluble carbohydrates and produce short-chain organic acids. It is a longstanding technology^1^ that has been revived in recent years^2–6^. In a study carried out in Brazil, Bernardes et al.^7^ mentioned that 52.4% of dairy farmers adopted grain silage (corn or sorghum) in the animals diets, 16.6% corresponded to rehydrated corn grain silage.

The use of this technology gained popularity mainly due to the obstacles commonly observed for high-moisture corn grain silage, for example, the narrow window for harvesting moisture grains. It also allows for strategic buying in times of low corn prices. Additionally, the storage of rehydrate grains as silage can eliminate or reduce the development of fungi and, consequently, avoid contamination by mycotoxins in the stored materials, as well as positive changes can be achieved in the nutritional value of the feed produced^2,8,9^.

During the fermentation process, microbial proteases, as well as organic acids produced by lactic acid bacteria, promote solubilization of the protein matrix that surrounds the starch granules, increasing digestibility by ruminal microorganisms^3,10^. However, rehydrated grain silage requires longer storage times to produce greater increases in dry matter digestibility^5,11^. Carvalho et al.^11^ reported an increase in in vitro dry matter digestibility of rehydrated corn grain silage at 280 days of storage compared to 30, 60, 90 and 150 days of storage. Increased in situ starch digestibility of rehydrated corn grain silage was observed by Fernandes et al.^12^ after 120 days of fermentation, compared to corn at ensiling, with values of 92.0 and 72.0%, respectively. For rehydrated sorghum grains silage, Santos et al.^13^ observed that cows fed diets containing silages stored for 30 days had lower starch digestibility (86.9%) compared to cows fed diets with silages stored for 90 days (89.3%).

Exogenous proteases are promising additives that promote nutritional improvements in corn plant silage^14^, high-moisture corn grains^15^, and rehydrated corn grains^16^, in shorter storage times, which ranged from 30 to 70 days depending on the type of silage and dose of enzyme used. Proteases are enzymes that hydrolyze peptide chains under suitable pH and temperature conditions, which, during fermentation, provide increases in the concentrations of ammonia and soluble protein in silages that present a positive correlation with starch digestibility, as reported by Ferrareto et al.^17^ and Kung et al.^15^.

To date, there are no reports in the literature on the use of exogenous proteases in the silage of rehydrated sorghum grains, and, in Brazil, with rehydrated corn grains. The hypothesis of our study is that the addition of exogenous protease to the silage of rehydrated corn and sorghum grains favors the solubilization of the protein fraction and improves the starch digestibility of the silages in a shorter storage time. Therefore, the objective was to evaluate the effects of exogenous protease addition on fermentation and the nutritional value of rehydrated corn and sorghum grain silages in different storage periods.

## Methods

### Location and silage preparation

The experiment was conducted at the Department of Animal Science of the Federal University of Viçosa (Federal University of Viçosa—UFV, Viçosa, MG, Brazil). Viçosa is located at 20°45′ south latitude, 42°51′ west longitude and 657 m above sea level, with a mean annual rainfall of 1.341 mm.

Fifty kilograms of corn (CG) and sorghum (SG) grains were obtained at the Federal University of Viçosa, UFV and subjected to milling in a hammer mill (DMP-2, Nogueiras, São João da Boa Vista, São Paulo, Brazil) with a 3-mm sieve. Then, the grain moisture was reconstituted to 35%, with the aid of a feed mixer for homogenization. The rehydrated corn and sorghum grains were distributed into 48 piles (24 CG piles and 24 SG piles), containing 2.5 kg of rehydrated grains in each pile.

Te current study complies with Brazilian ethical regulations. All methods were performed in accordance with relevant guidelines and regulations for plants.

The commercial product FoodPro PAL (PD 263063-3.0EN, DANISCO), a source of exogenous protease, has a composition of (w/w) 50% glycerin, 32–40% water, 10–12% aspergilopepsin I, and 0–2.8% sodium sulfate. Aspergilopepsin is a protease of fungal origin produced by *Aspergillus niger* that has optimal activity at pH 2.5 to 3.0 and a temperature from 55 to 60 °C, as specified by the manufacturer. The amounts of the commercial product referring to the doses evaluated in the study were diluted in 50 mL of distilled water and randomly applied to the piles. The same amount of water was sprayed on the control treatment (0% enzyme addition). The material was homogenized, and 1 kg of corn and sorghum grain was packed into nylon-polyethylene bags (25 × 35 cm; Doug Care Equipment Inc., Springville, CA), and the air was evacuated from the bags using a vacuum sealer (Eco vacuum 1040, Orved, Italy). Two bags were prepared in each pile, referring to 60 and 90 days of fermentation. The bags were stored in the laboratory at room temperature. The remaining material (500 g) from period zero was stored in polyethylene bags for further analysis. Before ensiling, samples of dried and rehydrated grains were collected for material characterization (see Supplementary Table S1).

A 2 × 6 × 3 factorial scheme was used, with 2 grains (corn and sorghum), 6 doses of the enzyme (0, 0.3, 0.6, 0.9, 1.2, and 1.5% based on NM) and 3 fermentation periods (0, 60, and 90 days) in a completely randomized design, with 4 replications.

### Fermentation profile

Water extracts from the silage or grain (day 0) samples were prepared by homogenizing 25 g of sample in 225 ml of sterile solution (Ringer Solution, Oxoid, Hampshire) in an industrial blender for 1 min. Then, the extract was filtered through a double layer of sterile gauze, and the pH was measured with the aid of a potentiometer (Tecnal, SP, Brasil).

A 15 mL aliquot of the extract was filtered through Whatman 54 filter paper (Whatman, Florham, NJ) and packaged in tubes containing 100 μL of H_2_SO_4_ 50%, for further analysis of ammoniacal nitrogen (NH_3_-N)^18^, water soluble carbohydrates (WSC)^19^, lactic acid (LA), acetic acid (AA), butyric acid (BA), and ethanol (ETA) by high-performance liquid chromatography (HPLC, Dionex Corporation, Sunnyvale, CA, USA)^20^.

### Microbial population

The populations of lactic acid bacteria (LAB), enterobacteria (ENT), molds, and yeasts in the grains before ensiling (day 0) and in the respective silages were quantified. An aliquot (10 mL) of the water extract (25 g of grain/225 mL of sterile saline solution) was subjected to serial dilution (10^–1^ to 10^–8^). Microorganisms were cultured in sterile Petri plates on De Man, Rogosa and Sharpe Ágar of LAB; Violet Red Bile of ENT and Potato Dextrose Ágar, supplemented with 1.5% of 10% tartaric acid (wt/vol), for molds and yeasts, using the pour-plate plating technique. The plates were incubated in an oven, with the temperature and period determined for each group of microorganisms as follows: ENT, 37 °C/24 h; LAB, 37 °C/48 h; yeast and molds, 25 °C/72 and 120 h, respectively. Plates with between 30 and 300 colony forming units were counted (cfu).

### Chemical composition and in situ ruminal digestibility

Samples of grains before ensiling and silages were dried in a forced ventilation oven at 55 °C for 72 h and then ground in a Willey mill with a 1 mm sieve. The DM (934.01 method) and crude protein, CP (984.13 method) contents were analyzed according to AOAC^21^, neutral detergent fiber (NDF) according to Mertens^22^, starch according to Hall^23^, and total soluble protein (P-sol) obtained after treating the samples with borate phosphate buffer (BPB), where the P-sol values corresponded to fractions A and B1, obtained from the difference between total nitrogen and insoluble nitrogen in BPB according to Licitra et al.^24^.

For the in situ ruminal starch digestibility assay (ISSD), an approximately 5.0 g sample (3 mm particle size) from each treatment was weighed individually into nylon bags (Sefar Nitex, Switzerland; porosity 50 μm, 400 cm^2^ of surface) and incubated in 4 Nellore cattle, with an average weight of 300 ± 18 kg, provided with a cannula in the rumen. After 7 h of incubation, the bags were removed from the rumen and washed in running water until the water ran clear. Then, they were kept for 72 h in a ventilated oven at 55 °C, followed by 2 h in an oven at 105 °C, and then weighed. The residue from each nylon bag was removed and ground in a knife mill (Tecnal, Piracicaba, São Paulo, Brazil) with a 1 mm sieve and placed in polyethylene bags for further analysis of residual starch according to Hall^23^.

Fifteen days before incubation, the animals used in the test received a diet containing 50% corn silage and 50% concentrate, on a dry basis.

The experimental procedures were approved by the rules of the Ethics Committee for the Use of Production Animals of the UFV (CEUAP/UFV, protocol nº 037/2018). The methods were also in accordance with Animal Research Reporting In Vivo Experiments (ARRIVE) guidelines for the reporting of animal experiments.

### Statistical analyses

The organic acid and ethanol data were analyzed in a 2 × 6 × 2 factorial scheme, in which the fermentation period 0 (zero) was not considered; the other variables were analyzed in a 2 × 6 × 3 factorial scheme, in a completely randomized design. The type of grain (G), the enzyme doses (E), and the fermentation periods (P), as well as the interactions between factors, were considered fixed effects according to the model:

1 $$Y_{ijkl} = \mu + {\text{G}}_{i} + {\text{E}}_{j} + {\text{P}}_{k} + \left( {{\text{GE}}} \right)_{ij} + \left( {{\text{GP}}} \right)_{ik} + \left( {{\text{EP}}} \right)_{jk} + \left( {{\text{GEP}}} \right)_{ijk} + {\text{e}}_{ijkl}$$

where, *Y*_*ijk*_ = response variable; µ = general constant; G_i_ = grain effect *i*; E_j_ = enzyme effect *j*; (GE)_*ij*_ = interaction of grain *i* and enzyme *j*; (GP)_*ik*_ = interaction of grain *i* and period *k*; (EP)_*jk*_ = interaction of enzyme *j* and period *k*; (GEP)_*ijk*_ = interaction of grain *i*, enzyme *j* and period *k*; and e_*ijkl*_ = random error assuming an independent normal distribution, NID (0,σ^2^). Following analysis of variance, the significant interactions were determined and the means compared using the Tukey test. The enzyme factor was analyzed using regression analysis, and the equations were chosen based on their coefficients of determination (R^2^) and the significance of the regression coefficients. The critical level of probability of a type I error adopted was 0.05, through the PROC MIXED of SAS version 9.4^25^. For the variables P-sol, NH_3_-N, ISSD, and NDF, the averages estimated by model (1) were adjusted to a quadratic polynomial model with a plateau response using the PROC NLIN procedure of SAS version 9.4, according to the equations:

$$Y = a + bx + cx^{{2}} ,\;{\text{se}}x < x_{0} \;\left( {{\text{quadratic}}} \right)$$

$$Y = p,\;{\text{se}}x \ge x_{0} \;\left( {{\text{plateau}}} \right)$$

where, *Y* = variable content as a function of dose *x* of enzyme; *p* = plateau; *a*, *b,* and *c* = estimated model parameters. Thus, for *x* values less than *x*_*0*_, the model describing the response *Y* is a quadratic function, and for *x* values greater than or equal to *x*_*0*_, the equation is a constant or plateau.

## Results

### Fermentation profile and microbial population

There was an effect (*P* < 0.01) of the G × E × P interaction on LA, NH_3_-N, and yeasts. The variables pH, ETA, and LAB were affected (*P* < 0.05) by the interaction G × E, G × P and E × P, while AA was affected (*P* = 0.02) only by E (Table 1).

**Table 1:** Effects of grain, enzyme, period and interactions for fermentation profile variables and microbial populations of rehydrated corn and sorghum grain silages.

Item	Probability^1^							SEM^2^
	G	E	P	G × E	G × P	E × P	G × E × P	
pH	< 0.01	< 0.01	< 0.01	< 0.01	< 0.01	< 0.01	0.15	0.09
Latic acid	< 0.01	< 0.01	< 0.01	0.29	0.19	0.11	0.008	0.70
Acetic acid	0.27	0.02	0.23	0.09	0.96	0.18	0.88	0.27
Ethanol	< 0.01	< 0.01	< 0.01	< 0.01	0.025	< 0.01	0.25	0.27
Ammonia N	< 0.01	< 0.01	< 0.01	< 0.01	< 0.01	< 0.01	< 0.01	0.38
LAB^3^	0.01	0.37	< 0.01	0.003	0.009	0.004	0.10	0.05
Yeast	< 0.01	< 0.01	< 0.01	< 0.01	0.02	< 0.01	< 0.01	0.06

^1^Probability of treatment effects: G = effects of grain, E = effects of enzyme, P = effect of period, G × P = interaction between grain and enzyme, G × P = interaction between grain and period, E × P = interaction between enzyme and period, G × E × P = interaction between grain, enzyme and period.

^2^SEM = mean standard error.

^3^Latic acid bacteria.

**Figure 1:**
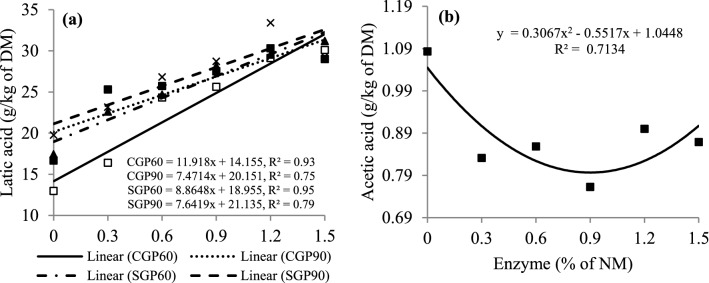
Regression analysis for lactic acid concentrations affected by G × E × P interaction (a, *P* = 0.0008, SEM = 0.705) and enzyme effect for acetic acid concentrations (b, *P* = 0.019, SEM = 0.271) in rehydrated corn and sorghum grain silage, treated or not, with enzyme in different fermentation periods. CG = corn grain; GS = sorghum grain; P = period (60 and 90 days).

There was a linear increase in LA concentrations (*P* < 0.01) with an increase in enzyme doses in CG and SG silages registering values of 32.03 and 32.25 g/kg of DM at 60 days and 31.36 and 32 0.60 g/kg DM at 90 days of fermentation at a dose of 1.5%, respectively (Fig. 1). The AA concentration observed in the silages was fitted to a quadratic model (*P* = 0.008) (Fig. 1).

CG and SG silages showed similar ETA concentrations, except at doses of 1.2% (*P* = 0.02) and 1.5% (*P* = 0.008) of enzyme, in which SG silages showed higher concentrations than CG (see Supplementary Fig. S1). The presence of BA was not detected in the silages evaluated in the present study, and no populations of enterobacteria or molds were observed in the silages.

At the time of ensiling, the SG had a higher (*P* < 0.01) LAB population, but at 60 (*P* = 0.34) and 90 (*P* = 0.67) days of fermentation, there was no difference compared to CG silages. In both silages, the highest (*P* < 0.01) LAB populations were recorded at 60 days of fermentation compared to periods 0 (zero) and 90 days (see Supplementary Fig. S2). SG silages showed higher yeast populations (*P* < 0.05) than CG silages at 60 and 90 days of fermentation, except silages with 0 and 1.2% doses of enzyme (see Supplementary Fig. S2).

**Figure 2:**
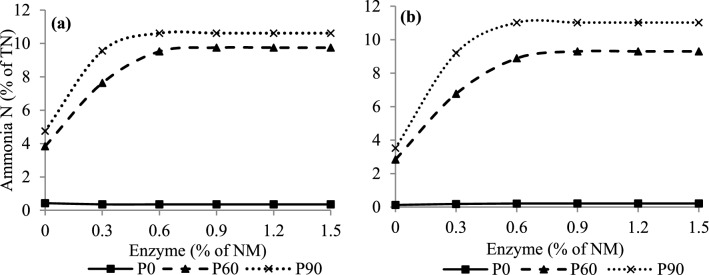
Concentrations of ammoniacal nitrogen in corn (**a**) and sorghum (**b**) grain silages rehydrated, treated or not, with enzyme in different fermentation periods. Corn grain (**a**) P0: Y = 0.428 − 0.55x + 1.02x^2^, se x < 0.27 e Y = 0.36, se x ≥ 0.27, R^2^ = 0.64, P60: Y = 3.84 + 15.86x − 10.63x^2^, se x < 0.75 e Y = 9.75, se x ≥ 0.75, R^2^ = 0.99, P90: Y = 4.75 + 22.52x − 21.57x^2^, se x < 0.52 e Y = 10.62, se x ≥ 0.52, R^2^ = 0.96. Sorghum grain (**b**) P0: Y = 0.13 + 0.24x − 0.17x^2^, se x < 0.70 e Y = 0.21, se x ≥ 0.70, R^2^ = 0.41, P60: Y = 2.84 + 16.10x − 10.03x^2^, se x < 0.80 e Y = 9.30, se x ≥ 0.80, R ^2^ = 0.98, P90: Y = 3.52 + 25.38x − 21.47x^2^, se x < 0.59 e Y = 11.02, se x ≥ 0.59, R^2^ = 0.97.

The data observed for the NH_3_-N concentration in CG and SG silages (Fig. 2) were fitted to a quadratic polynomial model with a plateau response. Increases in NH_3_-N concentrations were observed up to doses of 0.75% (*P* < 0.001) and 0.50% (*P* = 0.006) enzyme in CG silages and 0.80 (*P* = 0.002) and 0.59% (*P* = 0.004) in SG silages at 60 and 90 days of fermentation, respectively, with subsequent stabilization of NH_3_-N concentrations in silages (Fig. 2). There was the approximately 8.88 and 11.05 fold increases in NH_3_-N concentrations in CG silage; and 19.43 and 25.14 fold increases in SG silage at 60 and 90 days of fermentation, respectively (Fig. 2).

### Chemical composition and in situ ruminal digestibility

There was an effect of the interaction G × E × P on all variables of the nutritional value of the silages, except for DM, which was affected by the interactions G × P (*P* = 0.01) and E × P (*P* < 0.01), while CP was affected by the factors G and P (Table 2).

**Table 2:** Effects of grain, enzyme, period and interactions for variables of chemical composition and in situ starch digestibility of rehydrated corn and sorghum grain silages.

Item	Probability^2^							SEM^3^
	G	E	P	G × E	G × P	E × P	G × E × P	
Dry matter	< 0.01	0.08	< 0.01	0.07	0.01	< 0.01	0.07	0.74
Water-soluble carbohydrates	< 0.01	< 0.01	< 0.01	0.03	< 0.01	< 0.01	< 0.01	0.34
Crude protein	< 0.01	0.35	< 0.01	0.45	0.75	0.24	0.39	0.97
P-sol^1^	< 0.01	< 0.01	< 0.01	< 0.01	< 0.01	< 0.01	< 0.01	2.52
Starch	< 0.01	< 0.01	0.007	< 0.01	< 0.01	< 0.01	0.04	5.87
Neutral detergent fiber	0.01	< 0.01	< 0.01	0.002	0.003	< 0.01	< 0.01	2.49
In situ starch digestibility	< 0.01	< 0.01	< 0.01	< 0.01	< 0.01	< 0.01	< 0.01	19.91

^1^P-sol = Total soluble protein in borate phosphate buffer (BPB).

^2^Probability of treatment effects: G = effects of grain, E = effects of enzyme, P = effect of period, G × P = interaction between grain and enzyme, G × P = interaction between grain and period, E × P = interaction between enzyme and period, G × E × P = interaction between grain, enzyme and period.

^3^SEM = mean standard error.

The WSC concentrations observed for CG at the time of ensiling (P0) and in the silage at 60 days of fermentation were higher (*P* < 0.01) than those observed for SG at all enzyme doses (see Supplementary Fig. S3). A similar result was observed for starch concentrations, in which CG presented higher values (*P* < 0.05) than SG at the time of ensiling. At 60 and 90 days of fermentation, starch concentrations in CG silages were also higher, except at doses of 0% (zero), 0.6%, and 1.5% enzyme (see Supplementary Fig. S3).

**Figure 3:**
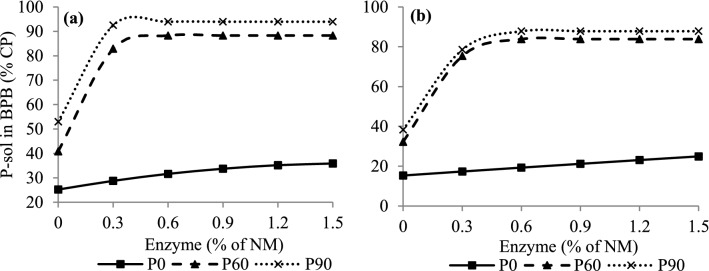
Concentrations of soluble protein (P-sol) in borate phosphate buffer (BPB) from corn (**a**) and sorghum (**b**) grain silages rehydrated, untreated and treated with enzyme in different fermentation periods. Corn grains (**a**) P0: Y = 25.22 + 13.04x − 3.96x^2^, if x < 1.65 and Y = 35.96, if x ≥ 1.65, R^2^ = 0.97, P60: Y = 40.83 + 209.39x − 230.95x^2^, if x < 0.45 and Y = 88.29, if x ≥ 0.45, R^2^ = 0.99, P90: Y = 52.99 + 222.32x − 301.75x^2^, if x < 0.37 and Y = 93.94, if x ≥ 0.37, R^2^ = 0.99. Sorghum grain (**b**) P0: Y = 6.3915x + 15.36, R^2^ = 0.80, P60: Y = 33.25 + 204.48x − 202.67x^2^, if x < 0.50 and Y = 83.83, if x ≥ 0.50, R^2^ = 0.99, P90: Y = 38.29 + 187.27x − 177.04x^2^, if x < 0.53 and Y = 87.81, if x ≥ 0.53, R^2^ = 0.99.

The CP variable was affected by the factors G (*P* < 0.01) and P (*P* < 0.01) (Table 2). SG silages showed higher values (*P* < 0.01) than CG silages, with values of 100.9 and 86.1 g/kg DM, respectively. At 60 and 90 days of fermentation, a lower value (*P* < 0.01) was observed for this variable compared to period 0 (zero), with values of 90.1, 90.2, and 100.1, respectively. Although exogenous protease treatment did not affect (*P* = 0.35) the CP concentration of rehydrated CG and SG silages, P-sol was affected (*P* < 0.01) by the G × E × P interaction (Table 2), whose data were fitted to a quadratic polynomial model with a plateau response, except for sorghum grain in the zero period (SGP0), which increased linearly with the enzyme dose (Fig. 3). Increases in P-sol concentrations were observed up to doses of 0.45% and 0.37% enzyme in CG silages, with plateau responses of 88.29% and 93.94% of CP, and 0.50 and 0.53% in SG silages, with plateau responses of 83.83% and 87.81% of CP at 60 and 90 days of fermentation, respectively (Fig. 3).

**Figure 4:**
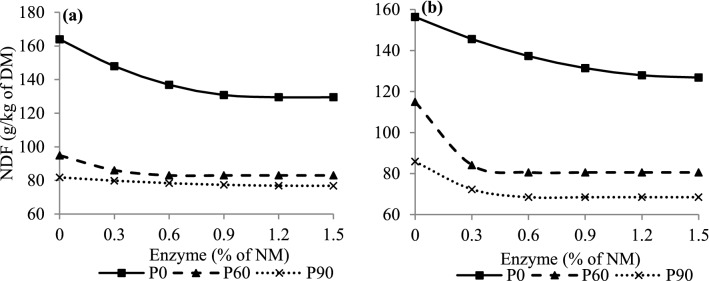
Neutral detergent fiber (NDF) concentrations of corn (**a**) and sorghum (**b**) grain silages rehydrated, untreated and treated with exogenous protease at different periods of fermentation. Corn grains (**a**) P0: Y = 163.97 − 61.82x + 27.71x^2^, if x < 1.12 and Y = 129.49, if x ≥ 1.12, R^2^ = 0.88, P60: Y = 94.89 − 39.24x + 32.35x^2^, if x < 0.61 and Y = 82.99, if x ≥ 0.61, R^2^ = 0.89, P90: Y = 81.81 − 7.44x + 2.77x^2^, if x < 1.34 and Y = 76.81, if x ≥ 1.34, R^2^ = 0.27. Sorghum grains (**b**) P0: Y = 156.33 − 39.76x + 13.39x^2^, if x < 1.48 and Y = 126.82, if x ≥ 1.48, R^2^ = 0.90, P60: Y = 114.95 − 155.69x + 176.19x^2^, if x < 0.44 and Y = 80.56, if x ≥ 0.44, R^2^ = 0.99, P90: Y = 85.86 − 61.79x + 54.86x^2^, if x < 0.56 and Y = 68.47, if x ≥ 0.56, R^2^ = 0.93.

An effect (P < 0.01) of the G × E × P interaction on NDF was observed (Table 2). The NDF data were fitted to quadratic polynomial models with a plateau response. It was observed that SG silage required lower enzyme doses at 60 and 90 days of fermentation to obtain a plateau response compared to CG silage (Fig. 4).

**Figure 5:**
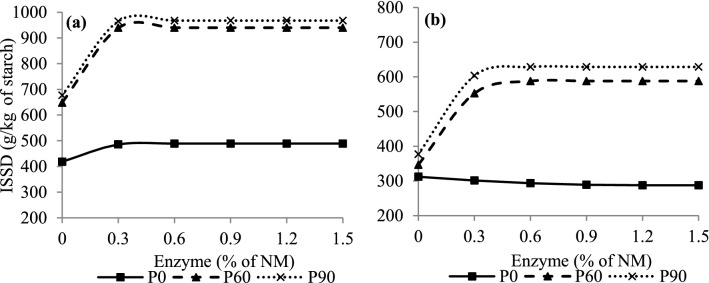
In situ starch digestibility (ISSD) of rehydrated, untreated and enzymatic corn (**a**) and sorghum (**b**) grain silages in different fermentation periods. Corn grains (**a**) P0: Y = 418.53 + 371.14x − 488.7x^2^, if x < 0.38 and Y = 489.001, if x ≥ 0.38, R^2^ = 0.83, P60: Y = 648.39 + 2003.25x − 3444.9x^2^, if x < 0.29 and Y = 939.62, if x ≥ 0.290, R^2^ = 0.99, P90: Y = 676.81 + 1767.55x − 2687.7x^2^, if x < 0.30 and Y = 967.424, if x ≥ 0.328, R^2^ = 0.99. Sorghum grains (**b**) P0: Y = 312.05 − 40.67x + 16.80x^2^, if x < 1.21 and Y = 287.43, if x ≥ 1.21, R^2^ = 0.14, P60: Y = 346.53 + 994.91x − 1025.4x^2^, if x < 0.49 and Y = 587.88, if x ≥ 0.49, R^2^ = 0.97, P90: Y = 376.99 + 1150.81x − 1316.1x^2^, if x < 0.44 and Y = 628.57, if x ≥ 0.44, R^2^ = 0.97.

For the ISSD variable, there was a G × E × P interaction (*P* < 0.01) (Table 2), and the data were fitted to a quadratic polynomial model with a plateau response at doses of 0.29% and 0.30% enzyme in CG silages and 0.50% and 0.44% in SG silages, which provided increases in starch digestibility of approximately 44.9% (648.39 *vs* 939.62 g/kg of starch) and 43% (676.81 *vs* 967.42 g/kg of starch) in CG silages and 69.6 (346.53 *vs* 587.88 g/kg of starch) and 66.7% (376.99 *vs* 628.57 g/kg of starch) in SG silages, at 60 and 90 days of fermentation, respectively (Fig. 5). Despite the greater increases in SG silages, CG silages presented numerically higher ISSD values (Fig. 5). Regardless of the fermentation period, based on the quadratic model of a plateau response, the dose of 0.30% enzyme provided maximum starch digestibility in CG silage, while for SG silage the starch digestibility reached a maximum value at doses of 0.50% and 0.44%, when stored for 60 and 90 days, respectively (Fig. 5).

## Discussion

The initial concentration of WSC has an important effect on the rate of pH decline during ensiling, as it is the substrate used for LAB metabolism. The low WSC concentrations of the grains before ensiling (see Supplementary Table S1) did not limit the growth of LAB (see Supplementary Fig. S2) or the fermentation process, since the pH values observed in the silages at days 60 and 90 of fermentation, which varied from 3.93 to 4.02, could be considered adequate for the evaluated silages (see Supplementary Fig. S4). The pH is directly affected by the concentration of organic acids produced by LAB, with LA being considered the most effective in reducing pH during fermentation^26^. The linear increase in LA concentrations, in our study (Fig. 1), was probably due to the fact that the addition of exogenous protease increased proteolysis and, consequently, provided greater release of peptides and free amino acids, which favor the growth of lactic acid-producing bacteria^16,27,28^.

The estimated minimum acetic acid value of 0.79 g/kg DM, at a dose of 0.9% of the enzyme (Fig. 1), can be considered adequate based on the recommended concentrations for this acid, in high-moisture corn grain silage, reported by Kung et al.^26^. An increase in AA concentration was reported by Young et al.^28^, in corn silage treated with exogenous protease at 150 days of fermentation. However, Kung et al.^15^ and Ferrareto et al.^16^ found no effect on the addition of protease in high-moisture corn grain silage or rehydrated corn grain silage, respectively.

In the present study, regardless of the dose and fermentation period, both silages presented ETA concentrations within the range (2–20 g/kg DM) proposed by Kung et al.^26^ for high-moisture corn grain silages. Yeasts are the main microorganisms responsible for producing ethanol during fermentation^27^ (see Supplementary Figs. S1 and S2).

The non-detection of enterobacteria at 60 and 90 days of fermentation was probably due to the pH of the ensiled mass, since this microbial group is sensitive to low pH, as suggested by Pahlow et al.^27^. As for the molds, during ensiling, these develop early in the process when there is still oxygen remaining inside the silo. However, during fermentation, the absence of oxygen associated with the presence of organic acids inhibits the growth of these microorganisms^29,30^, as observed in the present study. This demonstrates the effectiveness of epiphytic LAB (see Supplementary Fig. S2), which produce organic acids responsible for reducing pH^26^, in the fermentation process and controlling undesirable microorganisms during fermentation, such as enterobacteria and mold. Similar behavior was reported by Fernandes et al.^5^ in rehydrated corn and sorghum grain silages, which did not verify the presence of mold when the experimental silos were opened.

When forage and grains are ensiled, proteins are degraded naturally into peptides and free amino acids, and amino acid deamination can lead to an increase in NH_3_-N in the ensiled mass due to the action of microbial and plant enzymes^10,31^, this explain the increases in NH_3_-N concentrations in CG and SG silages at 60 and 90 days of fermentation (Fig. 2). Kung et al.^15^ suggested the inclusion of exogenous protease in high-moisture corn silage to increase proteolysis, which was previously seen as undesirable in the fermentation process^28^. The substantial increases in NH_3_-N concentrations in CG and SG (Fig. 2) silages confirmed the effectiveness of the exogenous protease used in the present study in the degradation of prolamins that surround the starch granules, making the starch more digestible by rumen microorganisms^32^. Thus, the concentration of NH_3_-N is a parameter indicative of proteolysis, which implies a possible improvement in starch digestibility, as it presents a positive linear correlation (*P* < 0.001) with digestibility^15,17^, which was confirmed in the present study (see Supplementary Fig. S5).

The lower DM content at 90 days of fermentation compared to 60 days, except for the 0.3% dose of the enzyme (see Supplementary Fig. S6), was probably due to microbial activity during the fermentation process, which naturally promotes reductions in DM contents with advancing ensiling time, as verified by Carvalho et al.^11^ and Da Silva et al.^3^ on CG silages and Santos et al.^13^ on rehydrated SG silage.

The amount of solubilized CP observed in our study indicates that proteolytic activity naturally occurred in CG and SG during fermentation, as reported by Hoffman et al.^32^ and Junges et al.^10^. However, this activity is increased with the addition of exogenous protease (Fig. 3), as observed by Kung et al.^15^ in high-moisture corn grain silage and by Ferrareto et al.^16^ in rehydrated GM silages. Therefore, based on the results of our study, it can be confirmed that P-sol is a parameter indicative of proteolytic activity, as it improves starch digestibility, showing a positive linear correlation with digestibility (see Supplementary Fig. S5) as highlighted Ferrareto et al.^17^ and Kung et al.^15^.

An interesting finding in our study was the reduction of NDF concentrations at 60 and 90 days of fermentation compared to period 0 (zero) with increasing doses of the enzyme in silages of both grains (Fig. 4). A possible explanation for this reduction would be the solubilization of cell wall components by the activity of acid-tolerant enzymes present in the ensiled grains^33^, which possibly contributed to the increase in WSC concentration throughout fermentation^34^ (see Supplementary Fig. S3). Young et al.^28^ also observed reductions in NDF concentrations in protease-treated whole corn plant silage as a function of storage time. Proteases can remove structural proteins in the plant cell wall, resulting in faster access to cellulose and hemicellulose by rumen microorganisms^35^, which probably also contributes to the increase in the P-sol concentrations of the silages treated in our study. These authors observed that the addition of protease to the ration before feeding the animals improved NDF digestibility.

Proteins that surround the starch granules in grains represent a physicochemical barrier to amylolytic microorganisms in the rumen, which limits the digestion of starch^32^. Kung et al.^15^ highlighted proteolysis as the main mechanism to increase the digestion of ruminal starch in silages with a starch source, because during fermentation, solubilization of the protein matrix occurs, which increases the contact surface for the action of ruminal microorganisms, as observed in our study and previous others^2,16,32^. The highest enzyme dose to provided maximum starch digestibility for SG silage observed in the present study (Fig. 5) is probably justified by the fact that the sorghum grain presents a higher proportion of proteins in the peripheral endosperm than in the corn grain, which increases resistance to water penetration, making it more resistant to enzymatic degradation^36^, thus providing lower P-sol compared to that of CG, both before and after fermentation. In addition, antinutritional factors may reduce intestinal and total sorghum digestibility in ruminants^37^.

It is worth mentioning that the stability of enzymes and their ability to interact properly with the target substrate is a factor that can provide inconsistent responses when using this additive in silage. Each enzyme has an optimal temperature and pH range, acting most efficiently when the conditions are close to ideal^38,39^. Therefore, the responses obtained with the use of the enzyme in CG and SG silages, under the present conditions, support our hypothesis that the addition of protease increases starch digestibility.

In conclusion, the addition of 0.30% exogenous protease at the time of CG ensiling and 0.50% in rehydrated SG favored the proteolytic activity during fermentation and provided an increase in in situ starch digestibility in a shorter storage time. A practical implication is that, although sorghum is an interesting option compared to corn, considering that it is at its lowest historical commercialization price, sorghum requires a dose of 66.6% higher than that of corn to maximize starch digestibility after 60 days of fermentation. This fact must be taken into account when choosing the type of grain to be rehydrated and ensiled. However, it is noteworthy that the CG silages presented higher ISSD values than the SG silage. There is a need to perform studies to evaluate the performance of ruminants fed with these silages, and the cost benefit of the addition of exogenous protease, at the time of ensiling.

## Supplementary Information

Supplementary Information.

## Data Availability

All data generated or analyzed during this study are included in this published article.
