# Supplementary material for: Effects of exogenous protease addition on fermentation and nutritive value of rehydrated corn and sorghum grains silages
Source: Sci Rep. 2023 May 5;13:7302. doi: 10.1038/s41598-023-34595-w (PMC10162983; doi:10.1038/s41598-023-34595-w)
Supplement: Supplementary file 1 — Supplementary Information. [file 41598_2023_34595_MOESM1_ESM.docx]

**Supplementary Table 1**. Chemical composition, pH and microbial population of dried and rehydrated corn and sorghum grains before ensiling.

| Item¹ | Dry | | Rehydrated | |
| --- | --- | --- | --- | --- |
|  | Corn | Sorghum | Corn | Sorghum |
| DM, g/kg of NM | 882.4 | 889.2 | 634.7 | 642.5 |
| CP, g/kg of DM | 92.0 | 107.1 | 92.3 | 106.4 |
| P-sol, % of CP | 24.2 | 18.78 | 24.74 | 14.3 |
| NDF, g/kg of DM | 145.2 | 158.1 | 165.7 | 153.9 |
| ADF, g/kg of DM | 34.2 | 39.9 | 33.6 | 39.6 |
| Starch, g/kg of DM | 698.9 | 673.7 | 737.5 | 589.0 |
| WSC, g/kg of DM | 17.1 | 12.1 | 15.8 | 11.1 |
| Ammonia N (% of TN) | - | - | 0.43 | 0.14 |
| pH | - | - | 6.18 | 6.47 |
| LAB, log cfu/g | - | - | 4.82 | 5.12 |
| ENT, log cfu/g | - | - | 4.67 | 5.17 |
| Yeast, log cfu/g | - | - | 4.49 | 4.97 |
| Moulds, log cfu/g | - | - | 5.11 | 4.19 |

¹DM = Dry matter, NM = natural matter, CP = crude protein, P-sol = Total soluble protein in BPB, NDF = neutral detergent fiber, ADF = acid detergent fiber, WSC = Water-soluble carbohydrates, TN = total nitrogen, LAB = lactic acid bacteria, ENT = enterobacteria.

**Supplementary Figure 1.** Least squares mean of the G × E interaction for ethanol concentrations (*P* = 0.002, SEM = 0.269) in rehydrated corn and sorghum grain silage, treated or not, with enzyme in different fermentation periods. CG: corn grain. Bars indicate SEM. Means followed by the same letter^a-b^, unfolding of the grains in each dose of enzyme, do not differ from each other (*P* > 0.05) by the F test.


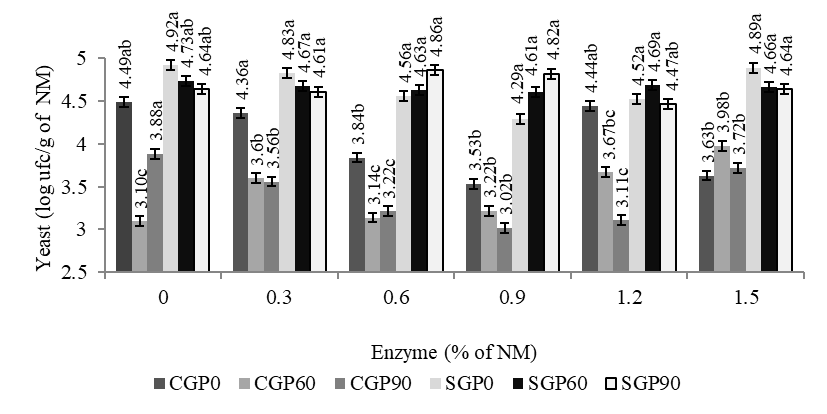

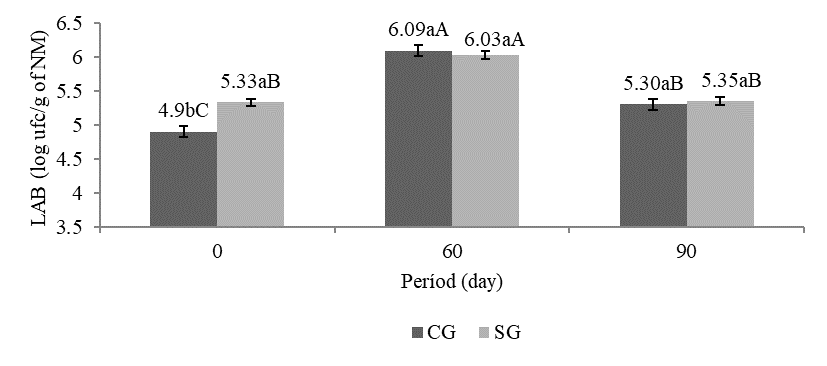


**(a)**

**(b)**

**Supplementary Figure 2.** Least squares mean of the G × P interaction for lactic acid bacteria population (LAB) (a, *P* < 0.009, SEM = 0.048) and G × E × P interaction for yeast population (b, *P* = 0.0015, SEM = 0.058) in rehydrated corn and sorghum grain silage, treated or not, with enzyme in different fermentation periods. CG: corn grain, SG: sorghum grain, P: period (0, 60 and 90 days). Bars indicate SEM. Figure a: Means followed by the same lowercase letter^a-b^, unfolding of the grains in each period do not differ from each other (*P* > 0.05) by the F test and means followed by the same uppercase letter^A-B^, unfolding of the period in each grain, do not differ between si (*P* > 0.05) by the Tukey test. Figure b: Means followed by the same letter^a-c^, grain unfolding and period in each enzyme dose, do not differ (*P* > 0.05) from each other by the Tukey test.

- **B**


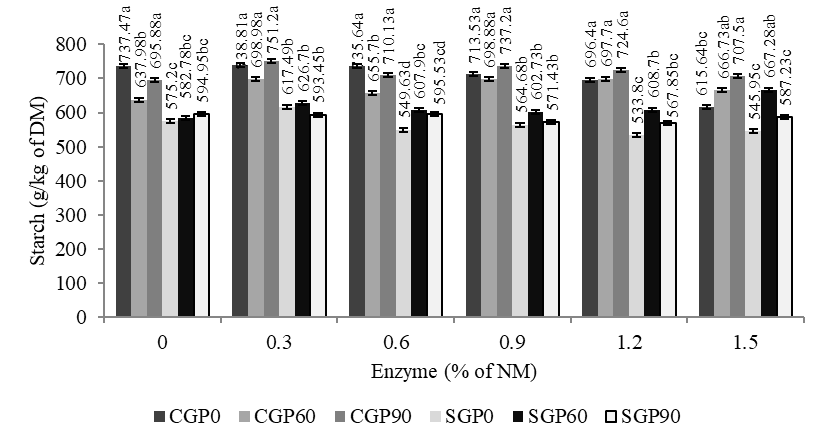

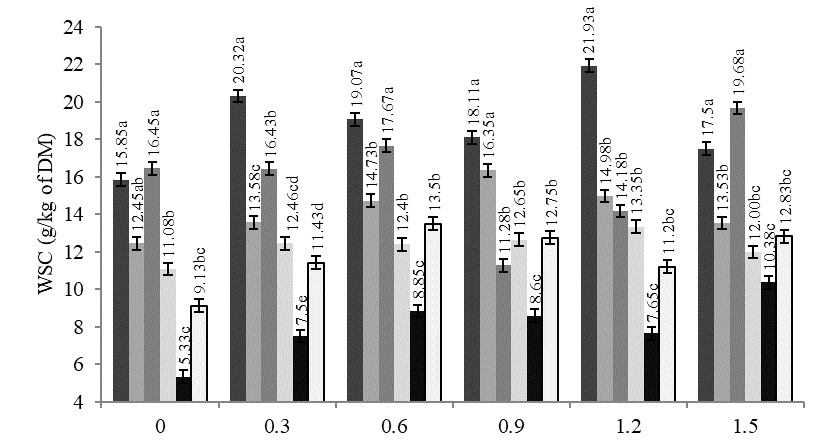


**(a)**

**(b)**

**Supplementary Figure 3.** Least squares mean of the G × E × P interaction for concentrations of soluble carbohydrates in water (WSC) (a, *P* < 0.0001, SEM = 0.338) and starch (b, *P* = 0.044, SEM = 5.873) in rehydrated corn and sorghum grain silage, treated or not, with enzyme in different fermentation periods. CG: corn grain, SG: sorghum grain, P: period (0, 60 and 90 days). Bars indicate SEM. Means followed by the same letter^a-d^, grain unfolding and period in each enzyme dose, do not differ from each other (*P* > 0.05) by the Tukey test.

**Supplementary Figure 4.** Least squares means for E × P interaction for pH values (*P* < 0.0001, SEM = 0.092) of rehydrated corn and sorghum grain silages, treated or not, with enzyme at different fermentation periods. Bars indicate SEM. Means followed by the same letter^a-b^, unfolding of the period in each dose of enzyme do not differ (*P* > 0.05) from each other by the Tukey test.


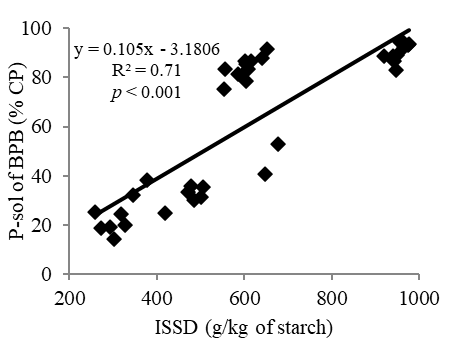

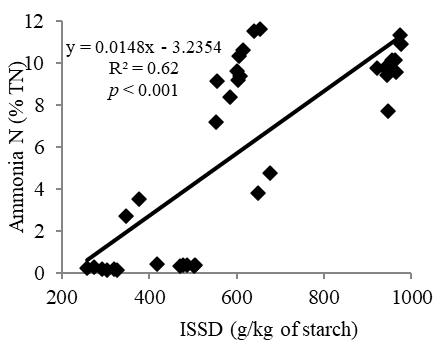


**(a)**

**(b)**

**Supplementary Figure 5.** Correlation between BPB-soluble protein (P-sol) (a), ammonia nitrogen (b) and in situ starch digestibility (ISSD) of rehydrated, treated corn and sorghum grain silages or not with enzyme, in different fermentation periods.

**Supplementary Figure 6.** Least squares mean of the E × P interaction for the DM content (*P* < 0.01, SEM = 0.740) in rehydrated corn and sorghum grain silage, treated or not, with enzyme in different fermentation periods. CG: corn grain, SG: sorghum grain, P: period (60 and 90 days). Bars indicate SEM. Means followed by the same letter^a-b^, unfolding of period in each enzyme, do not differ (*P* > 0.05) from each other by the Tukey test.
